# Ovary Development: Insights From a Three-Dimensional Imaging Revolution

**DOI:** 10.3389/fcell.2021.698315

**Published:** 2021-07-26

**Authors:** Bikem Soygur, Diana J. Laird

**Affiliations:** Department of Obstetrics, Gynecology & Reproductive Sciences, Center for Reproductive Sciences, Eli and Edythe Broad Center of Regeneration Medicine and Stem Cell Research, University of California, San Francisco, San Francisco, CA, United States

**Keywords:** ovary, folliculogenesis, tissue clearing, microscopy, 3D analysis

## Abstract

The ovary is an indispensable unit of female reproduction and health. However, the study of ovarian function in mammals is hindered by unique challenges, which include the desynchronized development of oocytes, irregular distribution and vast size discrepancy of follicles, and dynamic tissue remodeling during each hormonal cycle. Overcoming the limitations of traditional histology, recent advances in optical tissue clearing and three-dimensional (3D) visualization offer an advanced platform to explore the architecture of intact organs at a single cell level and reveal new relationships and levels of organization. Here we summarize the development and function of ovarian compartments that have been delineated by conventional two-dimensional (2D) methods and the limits of what can be learned by these approaches. We compare types of optical tissue clearing, 3D analysis technologies, and their application to the mammalian ovary. We discuss how 3D modeling of the ovary has extended our knowledge and propose future directions to unravel ovarian structure toward therapeutic applications for ovarian disease and extending female reproductive lifespan.

## Introduction

Female reproduction hinges on the development of the ovary, the organ which generates mature oocytes and produces hormones to regulate reproductive functions. The ovary harbors the oocyte reserve which supplies mature eggs for the duration of reproductive lifespan and concludes with the cessation of ovulation at menopause. The loss of ovarian function and subsequent drop in gonadal hormones has been associated with enhanced risk for cardiovascular disease (Atsma et al., 2006), osteoporosis, bone fractures (Van Der Voort et al., 2003), neurologic diseases, and adverse impact on quality of life compared to age-matched pre-menopausal controls (Broekmans et al., 2009; Parker et al., 2009; Rocca et al., 2009). Thus, the ovary may play a regulatory role in healthy aging beyond its indispensable function in female fertility.

The multifaceted functions of the ovary are accomplished by the coordination of diverse cell populations. The operational unit of the mammalian ovary is the follicle, which consists of an oocyte surrounded by granulosa and theca cells. The maturation of follicles from the non-growing or primordial stage toward large antral or ovulatory follicles is governed by signaling between follicles, within the supportive cells of the follicles, and the greater ovarian microenvironment (McGee and Hsueh, 2000; Da Silva-Buttkus et al., 2009). In addition, the function of follicles (and ultimately ovarian health) depends on hormones transported through blood vessels, signals received by nerve fibers, and homeostasis regulated by lymphatic vessels (Domínguez and Se, 2011; Brown and Russell, 2014). Understanding the dynamic and complex interactions between these different structures within the ovary has proven to be challenging.

In the past, the primary method for evaluating organs at the tissue and cellular level consisted of histological sectioning and immunostaining; however, three-dimensional, irregular, and dynamic biological structures are difficult to interpret by two-dimensional analysis. *In vivo* imaging methods such as ultrasound and tomography provide 3D visualization [reviewed in Feng et al. (2018) and Fiorentino et al. (2021)], however, only larger follicles can be detected. A newly developed imaging approach using optimized X-ray micro-Computed Tomography (microCT) detects secondary and later stage follicles (Fiorentino et al., 2020), however, imaging of small structures including primordial and primary follicles remains challenging. Three-dimensional (3D) imaging technologies were first developed to trace extensive nerve processes in the brain, which revealed the intricate structural and functional relationships (Dodt et al., 2007). 3D approaches have since been applied to structural and molecular features of developing as well as pathological organs. Initial 3D studies emphasized the importance of *in toto* analysis of ovaries (Cordeiro et al., 2015; Faire et al., 2015; Malki et al., 2015) however, the field of ovarian research has not widely adopted such approaches as fully as other organs. Despite the relatively small size of the ovary compared to other organs and ease of optical clearing, understanding of its 3D structure has lagged owing to its dynamic organization and extensive remodeling which occurs with each menstrual or estrus cycle.

The diagnosis of ovarian diseases is limited by our still evolving understanding of how the ovary changes during adulthood and undergoes aging. Defining the spatial relationships between components– including the vasculature, nerve fibers, and follicles– will enhance understanding of ovarian function and pathologies, and lay the foundation for identifying therapeutics for fertility preservation, ovarian cancer, and premature menopause. 3D modeling of the ovary can help demystify one of the earliest organs to undergo aging, which earned the title “canary in the coal mine” (Quinn and Cedars, 2017); apart from advancing reproductive medicine, the study of aging in the ovary may provide broader insight into the process of aging across and serve as a model of early detection for aging pathologies.

In this review, we outline the development and activity of mammalian ovarian compartments, discuss traditional methods of 2D ovarian analysis and their limitations. We summarize historical clearing techniques, recent advances and their facilitation of 3D imaging and quantitative analysis. Finally, we enumerate future directions for 3D analysis, specific applications to deepen understanding of ovarian development, as well as elucidate pathologic conditions of the ovary.

## Ovarian Compartments and Their Development

### Oocytes

Germ cell precursors in mice are segregated from somatic lineages around embryonic day 7.25 (E7.25) as a group of about 40 primordial germ cells (PGCs) located in the primitive streak near embryonic endoderm of the embryo (Ginsburg et al., 1990; McLaren, 2003). Diploid and sexually-undetermined PGCs transit through developing hindgut and dorsal mesentery before settling in the gonad primordium, or genital ridge, beginning at E10.5. PGCs expand by proliferation during their migration (Cantu et al., 2016), however, upon reaching the fetal gonads, their mitotic divisions involve incomplete cytokinesis, which leads to the formation of stable intercellular bridges that connect germ cells in cysts (Pepling and Spradling, 1998; Greenbaum et al., 2007, 2009). In the fetal ovary, extrinsic and intrinsic signals nudge germ cells in the anterior region to initiate meiotic machinery at ∼E13.5, which is followed by a wave-like propagation of meiotic initiation toward the posterior region (Menke et al., 2003; Bullejos and Koopman, 2004; Bowles et al., 2006; Koubova et al., 2006; Kumar et al., 2011; Arora et al., 2016; Vernet et al., 2020). As germ cells progress through different stages of meiotic prophase I (MPI), cyst breakdown begins, somatic support cells known as Granulosa cell precursors begin to encapsulate individual oocytes around E17.5. These structures, known as primordial follicles, are formed asynchronously and in a stereotyped pattern, beginning around birth at the center (or medulla region) of the ovary, followed by the outer surface (or cortex) and concluding by postnatal day 5 (PN5) (Pepling and Spradling, 2001; Pepling et al., 2010). The growth of follicles (to primary stage) similarly begins predominantly in the anterior-dorsal region of mouse ovaries around PN4 (Cordeiro et al., 2015) and continues thereafter, regulated by a combination of activator and suppressor signals that remains incompletely understood (Chen et al., 2020). Initial follicle growth continuously proceeds through stages that have been categorized morphologically as primary (a layer of cuboidal granulosa cells surrounds the oocyte) and secondary (multiple layers of granulosa cells and outer steroidogenic theca cells surround the oocyte); follicle death occurs at this stage until the onset of puberty, at which point the cyclic waves of follicle stimulating hormone and luteinizing hormone sustain the maturation of a subset of follicles to the antral stage (growing follicle with antral cavity). Although the default pathway for most antral follicles is atresia, a subset escape from that degenerative process. Among those, only dominant follicle(s) complete the final growth phase and get ovulated (McGee and Hsueh, 2000; Edson et al., 2009). After follicular rupture and ovulation, remaining supporting cells transform to progesterone-secreting cells and form the corpus luteum. Despite the foundational discoveries through decades of histological studies, the spatiotemporal dynamics and regulation of later follicle maturation, ovulation, and corpus luteum are still poorly understood.

### Somatic Cells

Ovarian fate depends on the differentiation and development of somatic lineages from bipotential precursors in the fetal gonads. These support cells eventually form what has been considered a supportive niche for the oocyte. The earliest gonad precursor cells originate from a thickening of the mesenchyme on the ventromedial side of the embryonic kidney (mesonephros), to which a cohort of coelomic epithelial cells migrate and proliferate; these two precursors establish the supporting steroidogenic and interstitial cells of the genital ridges [reviewed in Estermann et al. (2020)]. ‘Pre-granulosa’ cell populations emerge at different stages of ovarian development and contribute to the formation of distinct pools of follicles. The first group of pre-granulosa cells express *Foxl2* in early fetal stages, develop to granulosa cells in the medulla, and contribute to initial follicle formation during early stages of reproduction (Mork et al., 2012; Zheng et al., 2014). A second group of *Lgr5* expressing pre-granulosa cells are predominantly in the ovarian cortex and contribute to formation of cortical follicles (Ng et al., 2014; Rastetter et al., 2014; Niu and Spradling, 2020). Another somatic component of ovarian follicles, the theca cell layer, has two origins: from *Wt1*^+^ stromal cells of developing ovary and from *Gli*^+^ mesenchymal cells that migrate from the mesonephros (Liu et al., 2015). Signals from both granulosa cells and oocytes are required for the differentiation of theca cells to functional steroidogenic cells (theca interna) as well as to the fibrous, connective tissue layer (theca externa) (Liu et al., 2015).

### Vessel Network and Innervation

The vascular network transports gases, nutrients, and macromolecules required for the growth and maintenance of ovarian follicles. Blood vessels and lymphatics also remove metabolic waste and drain extracellular fluids from tissues, transporting proteins and lipids back to the bloodstream while controlling immune cell trafficking (Olszewski, 2003; Brown and Russell, 2014). Most organs do not undergo active remodeling of the vasculature, except during wound healing and pathological conditions such as cancers. However, the ovary is an exception, as dynamic changes occur in the vasculature during each hormonal cycle (Fraser, 2006). Time-lapse microscopy analysis and lineage tracing of *Tie2*-expressing endothelial cells revealed that small branches from the vessels at the border of the mesonephros and gonad extend into the primordial gonad at E11.5. With subsequent sex differentiation of the gonads, distinct vascular structures become evident in developing ovaries; without further endothelial cell migration from the mesonephros, blood vessels grow by branching morphogenesis, penetrate the developing ovaries and undergo local organization and remodeling within (Brennan et al., 2002; Coveney et al., 2008). In adult ovaries, anatomical and histological studies describe the morphology of the main vasculature entering the ovarian hilum and speculate that it functions to provide blood supply required for ovarian function; however, our knowledge of 3D vessel architecture and its relationship to normal ovarian function and diseases remains limited.

Peripheral nerves grow along blood vessels in most organs; however, this is not the case in the fetal ovary, as sympathetic nerves do not invade synchronously with vasculature, but rather innervate later than vasculature. Although neural projections in the medulla of fetal and adult mammalian ovary were identified through 2D histologic analyses (D’Albora et al., 2002; Dees et al., 2006), the spatial resolution of peripheral innervation together with vessel networks had to await the advent of 3D imaging and will be discussed later.

## Histologic Methods for Analysis of the Ovary

The field of anatomy was founded upon the meticulous description of macroscopic structures and inference of organ function. The dawn of the microscope moved anatomy into the cellular realm but necessitated the slicing of organs into thin pieces to view tissue structure. To parse most organs like the ovary, histologic approaches relied on sectioning specimens into 6 or 8 μm thick slices in meticulous order, staining with dyes or antibodies, and examining every fifth to every tenth section (Bucci et al., 1997; Flaws et al., 1997, 2001; Tilly, 2003; Sarma et al., 2020). Follicle quantification was carried out multiplying by a correction factor to the representative counts, which was not constant but changing based on the age of the ovary. This approach is labor intensive, and results vary considerably due to the accuracy of thickness of each slice, the number of slides counted, and the correction factor used in each study. Another technique to access follicle count is stereology, whereby objects of interest are counted in a known fraction of an organ (Gundersen, 1986; Gundersen et al., 1988). In this method, the ovary is uniformly and systematically cut into smaller pieces and a fraction of randomly-selected pieces are embedded, cut into thick (∼25 μm) sections, and follicles are counted by optical disector fractionator. In this algorithm, the raw count is multiplied by the inverse of the sampling fractions to calculate the total number of follicles in the ovary (Myers et al., 2004; Charleston et al., 2007; Sarma et al., 2020). Although stereology is deemed more accurate, the requirement for special equipment and expertise makes this technique less widely used.

With the development and implementation of machine learning technologies in biomedical sciences, quantitative analysis of various structures in biological samples can be expedited. New methods for automatic detection and counting of ovarian follicles on histological slides rely upon a deep learning algorithm known as Convolution Neuronal Networks (CNN) (Sonigo et al., 2018; Inik et al., 2019). Although machine learning provides unparalleled speed for analyzing samples, the information obtained at the cellular level derives from two-dimensional histological sections, which may be distorted or non-adjacent.

**FIGURE 1:**
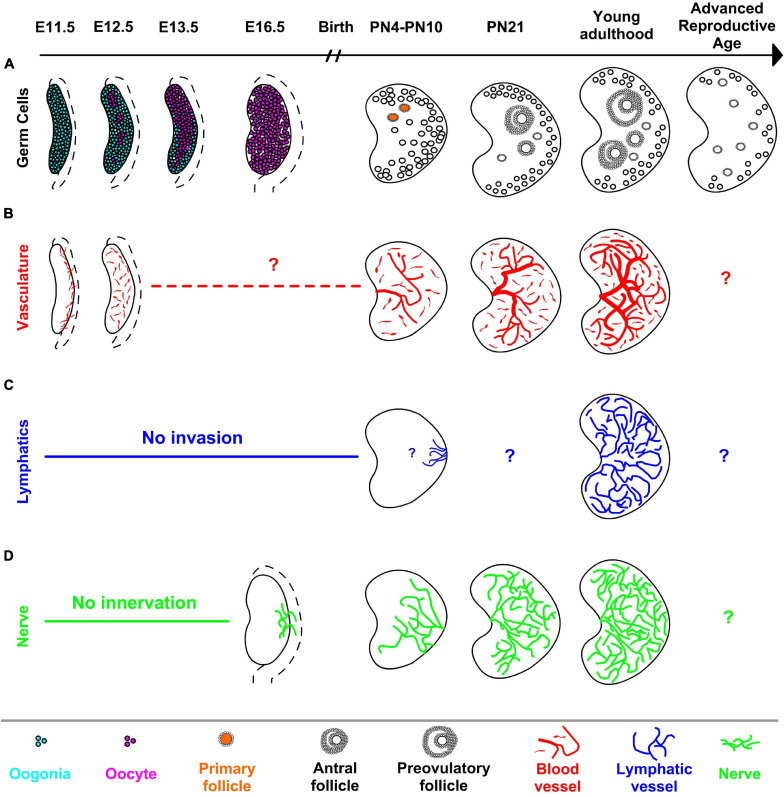
Main developmental events of the ovary organogenesis uncovered by 3D imaging in mice. **(A)** Following the localization in the gonad, germ cells undergo meiosis (in magenta) asynchronously starting as early as E12.5 in the anterior-medial region of the ovary. Germ cells located in the peripheral-posterior part of the ovary maintain their pluripotency (cyan) and initiate the meiosis later and by E16.5, all germ cells enter meiosis. Formation of primary follicles (in orange) and the first activation of follicular growth occur in the anterior-dorsal region of the postnatal ovary. While the number of follicles decreases with advanced maternal age, aged ovaries harbor mostly primordial and primary follicles due to the decreased number of later stage follicles. **(B)** Blood vessels (in red) at the gonad-mesonephros border expand into the gonad at E11.5 and proliferate to establish the blood vessel network of the developing ovary. Although further development of the vascular network in late fetal ovaries is unclear, blood vessels in the postnatal ovaries enter from the hilum and blood vessel diameters and branching increase by adulthood. **(C)** Lymphatic vessels (in blue) invade the ovary as early as PN10, however, the first point of contact inside the PN10 ovary is unclear with the resolution of micrographs. Similar to blood vessels, the adult ovary contains a complex network of lymphatic vessels. **(D)** Nerve fibers (in green) in the mesonephros innervate the ovary first from the dorsal side at E16.5, neural projections expand to medulla then cortex by PN4 and the neural network further increases with age. Dashed lines adjacent to fetal ovaries illustrate mesonephros. Question marks in **(B–D)** refer unidentified geography of related structures at given developmental points in 3D.

While spatial and temporal asynchrony of early ovarian development reflect the complexities of ovarian function and ultimately related diseases, the number of studies addressing spatiotemporal dynamics during later stages of ovarian development is limited by its opacity. In the fetal and neonatal mouse ovary, Cordeiro et al. (2015) used reporter proteins driven by stage-specific oocyte-specific promoters *Mvh*, *Gdf9*, and *Zp3* to reveal spatiotemporal dynamics of the first wave of folliculogenesis in 3D. *Mvh-*EGFP was detected earliest in the fetus, persisted in primordial follicles in postnatal ovaries, and extinguished in later stages of folliculogenesis. *Gdf9*-mCherry positive oocytes were detected starting at PN0. Zp3-AmCyan expression was first seen in oocytes located in the anterior-dorsal region of PN4 ovary, suggesting formation of primary follicles initially occurs in the anterior-dorsal part of the ovary (Figure 1A). Similarly, growing follicles were mainly detected in the dorsal region of postnatal ovaries. Although a powerful tool, endogenous reporter proteins must be visualized immediately after dissection, since the fluorescent signal drops dramatically after sample fixation. This constraint prevented optical clearing of the ovaries and limited analysis to structures positioned within 80 μm of the sample edge.

## Obstacles to Visualization of the Intact Ovary

3D imaging of whole embryos, organs and even adult bodies has exploded over the past decade due to advances in computational analysis, optical clearing, and image rendering. The challenges of combining macro and micro anatomy in intact organs include: (*1*) the need for appropriate microscopes and objectives with long working distances to accommodate large samples, (*2*) computation and storage required for big data sets, and (*3*) the opacity of most organs. The invention of confocal microscopy (Minsky, 1988), optical sectioning techniques (Conchello and Lichtman, 2005; Paddock and Eliceiri, 2014), and increased computational power, together with strategies for image data management (Wallace et al., 2015; Zhu et al., 2017; Bajcsy et al., 2018), overcame the first two challenges. In this review, we focus on the last: how opacity limits imaging of organs and how the ovary can be made transparent with different approaches.

**FIGURE 2:**
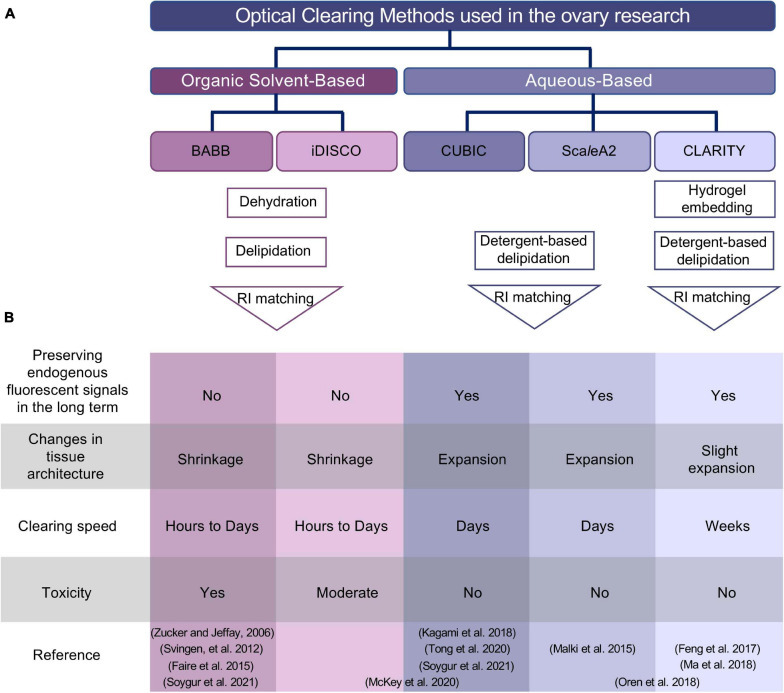
Summary of optical clearing methods applied to the ovary. **(A)** Outline of organic solvent-based and aqueous-based clearing approaches. Despite the use of different reagents in two different (BABB and iDISCO) organic solvent-based clearing methods, both protocols involve dehydration, delipidation, and a final RI matching step. Aqueous-based approaches (CUBIC and Sca*l*eA2) use high concentrations of detergents to remove lipids, while hydrogel embedding of the sample in CLARITY protocol maintains tissue and protein structure before incubation with high concentration of detergents to remove lipids. **(B)** Comparison of different methods based on experimental specifications. Studies used combined approaches are placed spanning the columns for iDISCO and CUBIC or Sca*l*eA2 and CLARITY in the reference column. BABB, benzyl alcohol and benzyl benzoate; iDISCO, immunolabeling-enabled three-dimensional imaging of solvent-cleared organs; CUBIC, clear, unobstructed brain/body imaging cocktails and computational analysis; CLARITY, clear lipid-exchanged acrylamide-hybridized rigid imaging/immunostaining/*in situ*-hybridization-compatible tissue hydrogel; RI, refractive index.

Similar to other organs, ovaries are composed of epithelial, connective tissues, vasculature and nerve fibers with varying components (e.g., membranes, nuclei, lipids, proteins, collagens, blood, tissue fluid, water etc.) that have different refraction indices, meaning that the light propagates through them at different speeds. Thus, light waves passing through the ovary scatter heterogeneously between different tissue layers, conferring an opaque or milky appearance. The underlying mechanisms of light scattering in biological samples and different clearing techniques are explained in detail by others (Richardson and Lichtman, 2015; Tian et al., 2021). To increase transparency, optical clearing methods aim to reduce the heterogeneity in refractive indices of different organ compartments by removing some tissue components (like lipids). This removal is performed in addition to immersing the sample in a solution with a refractive index more closely matched to the tissue or alternatively embedding it in hydrophilic polymers. Different clearing techniques are often combined, using active or passive removal of tissue components, followed by bathing the sample in refractive index-matched solution. Optical clearing methods were initially developed to elucidate complex neuronal circuits, brain structure, and function in the brain (Dodt et al., 2007). Later, the same techniques were applied to different organs, including ovaries; here, we summarize the underlying rationales, main advantages and drawbacks of those protocols in the field of ovarian research (Table 1 and Figure 2).

**TABLE 1:** Studies that applied different 3D visualization and analysis approaches in mouse ovaries.

	**Clearing reagent**	**Stage**	**Fixative**	**Immunolabeling applied (Y/N)**	**Microscope used**	**Structures analyzed**	**Quantitative analysis**	**References**
Organic solvent based	BABB	Embryonic, postnatal, and adult	4% PFA	Yes	Confocal microscope and optical projection tomography	3D geography of lymphatic and blood vessels	N/A	Svingen et al., 2012
	BABB	Embryonic	4% PFA	Yes	Confocal microscope	Spatiotemporal characteristics of meiotic initiation in germ cells	Imaris and Matlab	Soygur et al., 2021
	BABB	Postnatal	4% PFA and 1% Glut	No (stained with a fluorescent dye, LysoTracker)	Confocal microscope	Apoptotic granulosa cells in follicles	N/A	Zucker and Jeffay, 2006
	BABB	Postnatal and adult	MeOH:DMSO	Yes	Confocal microscope	The numbers and spatial distribution of follicles	Volocity and Matlab	Faire et al., 2015
	iDISCO	Adult	% PFA	Yes	Spinning disk confocal microscope	Growing follicles and interstitial compartment	N/A	McKey et al., 2020
Aqueous based	Sca*l*eA2	Embryonic	% PFA	Yes	Confocal microscope	Total germ cell numbers	Imaris	Malki et al., 2015
	CUBIC	Embryonic	% PFA	Yes (endogenous mCerulean, mCherry, and mOrange are also visualized)	Confocal microscope	Synchrony of meiotic onset and cytoplasmic sharing in germ cell clones	Imaris	Soygur et al., 2021
	CUBIC	Postnatal and adult	% PFA	Yes	Light-sheet microscope	Follicle numbers, 3D modeling of ovarian innervation and vasculature	Imaris	Tong et al., 2020
	CUBIC	Adult	% PFA	No (endogenous GFP signal is visualized)	Confocal and light-sheet microscopes	3D visualization of all cells in the ovary	N/A	Kagami et al., 2018
	CUBIC	Adult	% PFA	Yes (endogenous Tomato signal is also visualized)	Light-sheet microscope	Growing follicles, vasculature, and interstitial compartment	N/A	McKey et al., 2020
Hydrogel embedding	CLARITY	Postnatal, adult, and aged	% PFA	Yes	Confocal microscope	Dynamics of follicles, 3D structure and role of vasculature	Imaris and Matlab	Feng et al., 2017
	CLARITY	Adult	% PFA	Yes	Light-sheet microscope	Follicle numbers and vasculature	Imaris	Ma et al., 2018
Combined	iDISCO and CUBIC	Adult	% PFA	Yes	Light-sheet microscope and spinning disk confocal microscope	Growing follicles, oocytes, vasculature, and interstitial compartment	N/A	McKey et al., 2020
	CLARITY and Sca*l*eA2	Adult	% PFA	Yes (endogenous td Tomato signal is also visualized)	Light-sheet microscope	Blood and lymphatic vessels	Ilastik and Imaris	Oren et al., 2018

*PFA, paraformaldehyde; Glut, glutaraldehyde; MeOH, methanol; DMSO, dimethyl sulfoxide; N/A, not applied.*

## Clearing Techniques and Quantitative Approaches Used for Assessment of Ovaries in 3D

More than a century ago, Spalteholz (1914) developed the first clearing method by immersing large organs in the organic solvents benzyl alcohol and methyl salicylate. Although this method was detrimental to the tissue structure and only applicable for large samples (Steinke and Wolff, 2001), it formed the basis for almost all solvent-based clearing protocols to date.

### Imaging Oocytes and Follicles

#### Organic Solvent-Based Clearing

The main steps of solvent-based protocols are tissue dehydration, removing lipids, and impregnating the sample with clearing solution to homogenize the refractive index of remaining structures (Figure 2A). Zucker and Jeffay (2006) revived a solvent-based clearing approach by dehydrating late fetal mouse ovaries and immersing them in benzyl alcohol and benzyl benzoate (BABB). They stained intact fresh mouse ovaries with LysoTracker Red (LT) to label apoptotic cells. Subsequent fixation with 4% paraformaldehyde and 1% glutaraldehyde preserved tissue morphology as well as boosting background fluorescence signal that was advantageous for visualization of overall morphology. Imaging the intact ovary with a confocal microscope revealed that apoptosis occurs in individual granulosa cells or groups of granulosa cells located near an oocyte or antrum, which is the fluid-filled cavity in large follicles (Zucker and Jeffay, 2006). Although only fluorescent signal from dye and aldehyde-induced background signal were visualized, this first demonstrated that ovaries can be made transparent and visualized in 3D and provided a basis for future studies.

The first 3D visualization of oocytes *in situ* relied upon organic solvent-based clearing in BABB to render mouse ovaries transparent after immunostaining (Faire et al., 2015). Following fixation and permeabilization in Methanol:DMSO, immunolabeling was performed with nuclear markers NOBOX and GCNA to facilitate object segmentation, particularly for more densely packed primordial follicles. Based on the fortuitous observation that the oocyte nucleus increases in volume with follicle growth, primordial could be distinguished from growing follicles to produce the most accurate and direct quantification to date. Primordial follicles were counted in the intact ovary for the first time, showing a gradual drop in the total number from ∼5,000 at PN5 to ∼3,000 at PN7 and ∼800 at 6 months. Beyond quantification of the ovarian reserve, 3D imaging enabled analysis of the spatial organization of follicles revealing the precise borders of the medullary region in the neonatal ovary where the follicle growth starts.

In addition to advantages in quantitation and spatial analysis, whole-mount imaging is ideal for visualizing rare events. Given the controversy over oogonial stem cells, Faire et al. (2015) examined oocyte proliferation in the postnatal ovary by incorporation of the thymidine analog Bromodeoxyuridine (BrdU) and phospho-histone H3 (pHH3) immunostaining. However, coincidence of proliferation markers with NOBOX was exceedingly rare: 0.03% of oocytes at PN7. Proliferation markers could not be visualized during later ovarian development due to technical limitations. These results corroborate the definitive ovarian reserve and its decrease during aging. Although this study was the first to quantitatively analyze the intact ovary in 3D, the approach was limited in imaging, analysis, and technical capabilities. Ovaries older than 6-months could not be processed due to insufficient antibody penetration, clearing and/or elevated background. Accuracy was restricted to primordial follicles due to dim fluorescent signal of follicles at later stages, particularly antral. Finally, use of methanol as a fixative limited selection of antibodies since many epitopes are masked.

A recent study used 3D imaging to map the transition of germ cells in the fetal ovary from mitosis to meiosis (Soygur et al., 2021). Paraformaldehyde-fixed mouse ovaries were labeled with various nuclear markers and cleared in BABB. To determine the 3D distribution of germ cells, embryonic ovaries were computationally divided into seven segments along the longitudinal as well as transverse planes and germ cell numbers were quantified by using custom MATLAB scripts in Imaris analysis software. This revealed an earlier appearance of the earliest SYCP3+ meiotic cells as clusters in the core anterior ovary as early as E12.5. This radial geometry of meiotic initiation which precedes the anterior-posterior meiotic wave is orchestrated by intercellular bridges between developing germ cells (Figure 1A). Although the transition from mitotic to SYCP3+ leptotene and zygotene stage occurs asynchronously in a spatially-defined pattern, progression to the SYCP1+ pachytene stage was by contrast observed to be rapid and synchronous, suggesting that the time spent in the early stages of MPI differs amongst germ cells. By taking advantage of the well-established solvent-based clearing method, this study revealed spatiotemporal differences between critical developmental events in germ cells in mice, the functional consequence of which remains to be tested.

Although BABB was the first organic solvent used to clear mouse organs, it was not sufficient to clear bigger samples and alcohol-based dehydration rapidly quenched fluorescence signals. These limitations were overcome by replacing BABB with the combination of dibenzyl ether (DBE) and tetrahydrofuran (THF), known as 3DISCO solvent-based clearing (Erturk et al., 2012, 2014), or iDISCO in combination with immunolabeling (Renier et al., 2014). A major advantage of this method is the preservation of fluorescent signal longer, in addition to clearing large tissues, notably a whole adult mouse body with ultimate DISCO (uDISCO) (Pan et al., 2016). This protocol was improved with shortened processing times and increased fluorescent preservation by combined adjustment of temperature and pH in DISCO with superior fluorescence-preserving capability (FDISCO) (Qi et al., 2019). Among these variations, iDISCO was used to clear adult ovaries, achieving efficient fluorescent signal as well as successful antibody penetration to visualize follicular structures and the ovarian interstitial compartment in adult mice (McKey et al., 2020).

#### Aqueous-Based Clearing

Despite advances in organic solvent-based clearing techniques, the instability of endogenous fluorescent signals that resulted from the removal of water molecules during dehydration created a need for aqueous-based clearing approaches. Sucrose (Tsai et al., 2009), fructose/thioglycerol (SeeDB) (Ke et al., 2013), and formamide (ClearT) (Kuwajima et al., 2013) are aqueous solutions with high refractive indices that approximate that of tissues. Simple immersion in such aqueous clearing solutions preserves lipid structures, however, it is not sufficient to clear thick samples containing connective tissue such as ovaries. Sca*l*eA2 is an aqueous-based clearing approach that uses detergents instead of hydrophobic solvents to remove lipids while maintaining tissue hydration with urea and glycerol (Hama et al., 2011) (Figure 2A). Samples in Sca*l*eA2 undergo expansion due to hyperhydration, however, transparency is improved with a lower cost of reagents compared to some other aqueous-based solutions. Sca*l*eA2 was combined with sucrose preincubation to image oocytes with TRA98 in fetal ovaries during meiosis and fetal oocyte attrition (Malki et al., 2015). After manually correcting the image segmentation for a representative single z stack, the actual percentage of the error of automatic germ cell detection was calculated as 9–14%, which can be further improved with higher quality image acquisition. The total germ cell number decreased about 27% between E15.5 and E18.5, which is less than the almost twofold loss reported by histological sampling (Malki et al., 2014). Sca*l*eA2 with TRA98 immunofluorescence was also used to study right/left ovarian asymmetry. Earlier studies showed inequalities between right and left ovaries in function as the ovulation rate in mice (Wiebold and Becker, 1987). Similar to results presented by Faire et al. (2015) in adult ovaries, precise analysis of germ populations demonstrated no significant difference between right and left ovaries at E15.5 and E18.5. The work of Malki et al. (2015) laid the groundwork for 3D analysis of embryonic ovaries by providing a detailed analysis pipeline, error rate of the technique, and direct comparison between sampling and whole-mount methods.

Even greater transparency with aqueous clearing came from a chemical screen of mouse brain based on components of Sca*l*eA2. The Clear, Unobstructed Brain/Body Imaging Cocktails and Computational analysis (CUBIC) protocol (Susaki et al., 2014, 2015) can be applied as simple immersion or perfusion. The first reagent contains polyhydric alcohol (Quadrol), detergent (Triton-X 100), and urea and removes lipids. The second reagent, consisting of triethanolamine polyhydric alcohol, sucrose and urea, matches the refractive index of the tissue and provides improves transparency over Sca*l*eA2and preservation of endogenous fluorescent signals (Figure 2). CUBIC has been used to understand 3D structures of ovaries using endogenous fluorescent reporter proteins and immunolabeled structures. A modified CUBIC protocol was employed by Kagami et al. (2018) to visualize ubiquitous EGFP in adult mouse ovary as a proof of principle. Incubation in Sca*l*eA2-CUBIC-1 solution alone sufficiently cleared fetal mouse ovaries for visualization of endogenous fluorescent signals and immunolabeling (Soygur et al., 2021). 3D imaging of inducible multicolored reporters revealed clonal structure of germ cells; during cyst formation, 3D analysis provided quantitative evidence that cytoplasmic sharing occurs via germ cell intercellular bridges. While CUBIC clearing is sufficient to visualize endogenous proteins in fetal ovaries, McKey et al. (2020) reported that it did not provide optimal results in adults. However, this discrepancy may be attributable to minor differences in clearing protocols in these two studies. Combining two clearing approaches, iDISCO and CUBIC, improved clearing efficiency and allowed imaging of various cell populations: follicles, vasculature, interstitial cells, and neurons in adult ovaries (McKey et al., 2020). While this study is the first to combine two different clearing methods to visualize immunolabeled structures in adult mouse ovaries, immunolabeled structures could not be analyzed quantitatively in adult ovaries due to high background or inaccurate segmentation of image analysis software.

#### Hydrogel Embedding

Hydrogel embedding of tissues is used with aqueous clearing methods to help maintain biological/cellular structures through providing structural support. In the method dubbed CLARITY (Clear Lipid-exchanged Acrylamide-hybridized Rigid Imaging/Immunostaining/*in situ*-hybridization-compatible Tissue hYdrogel) (Chung et al., 2013), hydrogel monomers are infused during tissue fixation, and the polymerization of hydrogel mesh is initiated with heat to form a tissue-hydrogel hybrid. Subsequently, lipids are removed from the tissue-hydrogel hybrid with highly concentrated detergents, either passively or aided by electrophoresis, before immersing the tissue in a refractive index-matched clearing solution (Figure 2A). Feng et al. (2017) implemented a passive CLARITY protocol to render mouse ovaries transparent over 4–8 weeks. They were able to quantify follicles and their spatial distribution throughout development and aging (from PN3 to 12-months old). The volume of follicles increased ∼3 × 10^5^-fold from primordial to pre-ovulatory follicle whereas, follicle roundness decreased, reflecting massive follicle growth and dynamic tissue remodeling in the ovaries (Feng et al., 2017). Spatial analysis of follicle distribution within the ovary demonstrated that follicles tend to localize toward the center as folliculogenesis progresses (from primordial to antral follicles) and ovary undergoes active remodeling at each cycle as observed previously (Hirshfield and DeSanti, 1995). Within the ovary, follicles aggregated with similarly staged follicles, however, pre-ovulatory follicles had fewer primordial, primary, and secondary follicle neighbors compared to later follicular stages, suggesting that estrogen or other factors secreted by pre-ovulatory follicles may inhibit earlier stage of folliculogenesis.

### Imaging Vasculature in the Ovary

In addition to changes in the organization of follicles, whole-mount imaging revealed the dynamics of the vasculature in mouse ovaries. Organic solvent-based clearing was applied to mouse ovaries that express GFP under *Prox1* promoter in order to characterize the spatiotemporal development of the lymphatic network in the ovary (Svingen et al., 2012). *Prox*1-EGFP ovaries were fixed in 4% paraformaldehyde, immunolabeled with GFP and lymphatic vascular markers followed by embedding in agarose. BABB cleared ovaries were imaged with confocal microscope or optical projection tomography, and visually presented by using Imaris software. Previously, the presence of lymphatic vessels was reported in ovaries just before birth by *X*-gal staining of *Prox1*^+/lacZ^ mice (Brennan et al., 2002) however, 3D visualization of lymphatic vessels in *Prox*1-EGFP ovaries at higher magnification revealed that the first lymphatic vessels invade the ovary at PN10. This later development of ovarian lymphatics contrasts with other adjacent structure such as the uterus and ovarian ligaments in which lymphangiogenesis occurs during fetal development. 3D visualization of the lymphatic network using Prox1-GFP revealed expansion through the ovarian medulla and cortex during adulthood (Figure 1C). The lymphatic network mostly overlapped with endoglin-positive blood vessels, but by contrast, LYE1-positive small lymphatic capillaries were predominantly localized in ovarian and extraovarian rete. Enabled by 3D imaging and clearing, this first qualitative description of the complex network of lymphatic vessels (Svingen et al., 2012) lays the foundation for future studies to probe function of lymphangiogenesis by examining interactions with follicles in healthy, advanced age, as well as diseased ovaries.

CLARITY also enabled the mapping of dynamic changes in ovarian blood vessels during folliculogenesis, which would be difficult or impossible to visualize with traditional histology. Work by Feng et al. (2017) revealed that the largest vasculature originating from ovarian hilum was expanded 40% from PN3 to adulthood. At the follicle level, PECAM1+ endothelial cells were absent or sparse in primordial and primary follicles but became prominent in the thecal layer of secondary follicles, and remained through the antral, preovulatory follicles and the corpus luteum. Inducing follicular growth by hormone treatment at PN21 dramatically increased the diameter of the main vessel and revealed a structured organization of follicles around main vascular tree (Figure 1B). In mice with a heterozygous deletion of vascular endothelial growth factor A (*Vegfa*^+/−^), the same hormone treatment contributed to defects in ovarian vasculature, and ultimately decreased ovarian weight and reduced number of oocytes ovulated compared to wild-type; this functionally demonstrated the dependence of follicle maturation and successful ovulation upon vascular remodeling.

Further driven by the need to visualize the elaborate structure of the vascular systems, CLARITY and Sca*l*eA2 were combined to establish the Whole Organ Blood and Lymphatic Vessel Imaging (WOBLI) technique (Oren et al., 2018). This optimization replaced the costly FocusClear clearing reagent in the CLARITY protocol with Sca*l*eA2 to improve efficiency of tissue clearing in combination with immunolabeling. For high-resolution 3D structural resolution of blood vessels in the ovary and other organs, transgenic mice expressing tdTomato under the *Ve-cadherin* promoter were processed by WOBLI and imaged with light sheet microscopy. Although immersion of the sample in WOBLI clearing solution expanded the tissue (∼1.7- 2-fold), the broad morphology and follicular structures were maintained in the ovary. In pubertal mice, this protocol allowed for quantification of total vessel length, vessel diameter, vessel straightness and total number of branching points within the ovary (Oren et al., 2018). As blood vessels and lymphatics are responsible for homeostasis and immune function, 3D mapping of their dynamics provides valuable information toward targeted drug delivery for ovarian cancers or fertility treatment.

### Imaging Peripheral Nerves in the Ovary

The Capel group first charted peripheral innervation in the developing and neonatal ovary and using whole-mount imaging with combined iDISCO and CUBIC clearing techniques (McKey et al., 2019). Detailed 3D analysis of the intact gonad and adjacent mesonephros uncovered the spatiotemporal dynamics of innervation of the ovary and its striking sexual dimorphism with the developing testis. Immunofluorescence revealed that the neuronal network in the ovary is established by TH+ projections in the mesonephros that first invade the dorsal side of the ovary before birth, reaching the ventral side by passing through medulla (Figure 1D). While the innervation of the ovary begins during fetal development, the testis is recalcitrant to neural projections at the same timepoint, is likely due to repressive signals which are upregulated during male but not female somatic differentiation. Although this study was the first to identify patterns of innervation of the developing ovary, functional interactions between nerve fibers and somatic cells of the ovary during fetal development remain to be tested.

In a recent 3D study of innervation in postnatal and adult mouse ovaries using CUBIC-clearing Tong et al. (2020) showed that each follicle is innervated by a single neural fiber; furthermore, peripheral nerve branching around follicles increases at later stages of folliculogenesis (Figure 1D). They further showed that PMSG-induced follicular growth contributed to a visible increase in TH-positive neuronal fibers in the ovary within 48-h. Tracing the long and circuitous routes of nerve fibers could not be accomplished through traditional histology, but is ripe for 3D techniques. The growth and regeneration of nerve fibers in the ovary warrant further study, particularly focused on various pathologies related to neural network of the ovary.

## 3D Modeling of the Aging Ovary

Although the age-related decline in ovarian function has long been recognized, the structural and functional changes occurring in the ovary during aging are not well defined. Feng et al. (2017) applied the CLARTIY protocol to visualize 12-month-old mouse ovaries and quantified follicle numbers by using Imaris software. Consistent with prior reports, they demonstrated a decrease in the number of total follicles at this age of reproductive senescence, however, the percentage of primordial and primary follicles was found to increase in aged ovaries due to decreased numbers of secondary and later stage follicles (Feng et al., 2017). In addition to diminished follicle numbers, other studies have described dramatic changes that occur in the ovarian microenvironment with aging, and their potential impact ovarian function and follicle development (Duncan et al., 2018). More recently increased ovarian stiffness was associated with elevated collagen and decreased hyaluronan in 2D histological examination of aged ovaries (Amargant et al., 2020). Further insight into tissue properties in the ovary came from a 3D analysis of adult mouse ovaries which demonstrated that sphericity of follicles decreased in later stages of folliculogenesis due to the changes in ovarian rigidity; interestingly this change was not observed when folliculogenesis was artificially induced by hormone treatment in prepubertal mice (Feng et al., 2017). This result suggests that changes in the ovarian stroma over time regulate the stiffness of the ovary and affect the shape of follicles. Possible functional outcomes of altered physical properties of follicles remain to be tested.

Conventional histological analysis showed increased accumulation of extracellular matrix, particularly collagen I and III, leads fibrosis in the stroma with aging. Age-related fibrosis appears to be connected with infiltration of multinucleated macrophage giant cells in ovaries at advanced aged (Briley et al., 2016). Detailed transcriptome analysis revealed changes in the macrophage populations in aged ovaries that may contribute to the aging-associated chronic inflammation (known as inflammaging) in the ovaries (Zhang et al., 2020). Despite the detailed characterization of inflammation in ovarian aging, the location and dynamics of immune cells in ovaries has not been reported. 3D imaging will help to define the hallmarks of ovarian aging as well as the changes in tissue structure (follicles, vasculature, and nerve fibers) caused by age-induced fibrosis and inflammation.

## 3D Analysis of the Ovary in Different Species

The majority of 3D analysis techniques were initially developed for studies in mice because they are widely used mammalian models for human physiology and disease, while their small size increases clearing efficiency. Improvements in clearing and imaging technologies have allowed researchers to image intact human embryos as well as fetal organs. Using the 3DISCO protocol, gestational week 10.5 ovaries and the surrounding extensive vascular network were successfully visualized for the 3D human cell atlas (Belle et al., 2017).

More recently, a number of studies applied 3D modeling to different species to study ovarian diseases in rats. Ma et al. (2018) used CLARITY and 3D imaging to investigate follicle dynamics and the vessel structure in rat ovaries following the induction of Polycystic Ovary Syndrome (PCOS) by 5α-dihydrotestosterone (DHT). Despite the similar follicle numbers in both groups during the early stages of folliculogenesis, pre-ovulatory follicles were completely absent in PCOS-like ovaries. The authors performed low-frequency electro-acupuncture (EA), which positively increases ovulation and pregnancy success rates in humans with PCOS (Smith et al., 2016), and showed a quantitative restoration pre-ovulatory follicles in PCOS-like rats. In addition to the poor ovulatory response, the vascular architecture in PCOS-like rat ovaries was impaired. EA treatment partially rescued the PCOS-like phenotype by increasing total vascular area and volume, particularly in mature follicles, suggesting that elevated blood flow allows mature follicles to receive signals required for ovulation. The role of innervation in PCOS pathology and its possible treatment via EA therapy were also studied in rat ovaries using CUBIC clearing and 3D analysis (Tong et al., 2020). EA treatment reversed the increased innervation in the ovarian stroma and the diminished neuronal network around individual follicles in PCOS-like ovaries. Denervation studies suggested that EA treatment of this model of PCOS in rats is regulated through the superior ovarian nerve (SON). The 3D techniques applied in these studies uncovered intricate relationships between follicles, vasculature and nerve fibers in PCOS, which affects as many as 18% of women during their childbearing years (Ding et al., 2017). The expanded use of 3D analysis will grant more comprehensive view of PCOS as well as other complex pathologies.

Clearing and 3D imaging of ovaries in fish has proved to be challenging due to the high concentration of lipids in teleost oocytes. A recent study compared several clearing methods in medaka ovaries: (*i*) simple immersion in high-refractive index matching solutions, (*ii*) iDISCO, (*iii*) CUBIC, (*iv*) Ethyl cinnamate ‘ECi,’ and (*v*) combined CUBIC and ECi ‘C-ECi’ (Lesage et al., 2020). Solvent-based ECi and iDISCO techniques provided better transparency for paraformaldehyde-fixed medaka ovaries however, methyl green counterstaining produced high background with ECi clearing. To reduce the background staining that potentially arose from the remaining lipid in ECi-cleared ovaries, the authors varied pH, temperature, and combined CUBIC and Ethyl cinnamate ‘C-ECi’ techniques. Among all parameters tested, the combined C-ECi protocol (pH7, 50°C) best preserved the methyl green fluorescent signal. 3D analysis showed that medaka ovaries predominantly contain small follicles whereas post-vitellogenic (maturation) stage follicles comprise 2,3% of total follicles and localize on the ventral side of the ovary. Direct comparison of follicle numbers determined by 3D versus 2D analysis revealed that the prior 2D estimations lead to overcounting of intermediate follicles and undercounting of small and larger follicles. The same analysis pipeline was also applied to trout ovaries that contain bigger oocytes, yielding a similar predominance of small follicles. This study by Lesage et al. (2020) serves as a guide for clearing and analysis of ovaries with high lipid content and guide for future developmental studies in various species.

## Future Directions

3D visualization and analysis of the ovary at single cell resolution has great potential to reveal higher-order biological structure, while also holding promise for improving *in vitro* approaches. Until a few years ago, the generation of mature oocytes by *in vitro* reconstitution of oogenesis was a major challenge in the field of reproductive biology. A major breakthrough came with the recognition that mouse embryonic stem cell-derived primordial germ cell-like cells (PGCLCs) could be induced to initiate meiosis by co-culture with fetal ovarian somatic cells, which lead to the formation of reconstituted ovaries (rOvaries) and ultimately the generation of healthy offspring from these *in vitro* derived oocytes (Hikabe et al., 2016). While this model offers a unique platform for studying the regulatory mechanisms of oogenesis and paves the way for future human-focused studies, the relatively low efficiency of *in vitro* oocyte derivation would likely be improved by studies aimed at understanding the complex interactions between the oocyte and its niche (supporting cells, vasculature, and neuronal) in 3D. The comprehensive view of the ovary provided by 3D analyses highlights the dynamic changes occurring during development and will inform the roadmap for generating oocytes outside of the body (Table 2).

**TABLE 2:** Possible future applications of 3D imaging.

**What is known**	**Current unknown**	**Questions that can be answered using 3D visualization**
Mature oocytes can be generated by co-culturing mESCs-derived PGCLCs and fetal ovarian somatic cells (rOvaries) *in vitro*.	Why is the efficiency of *in vitro* oocyte derivation relatively low and how can it be improved?	Identifying *in vivo* oocyte-niche (supporting cells, vasculature, and neuronal) interactions in 3D and comparing 3D architecture of rOvaries with *in vivo* structure can lead to the development of better culture systems (for instance, recapitulating *in vivo* development by enrichment of the vasculature and neuronal progenitors in the culture systems).
Ovarian cancer organoids are generated by using patient-derived tumor cells to study the progression and treatment of ovarian tumors.	How closely do ovarian organoids mimic 3D structure of *in vivo* ovarian cancers?	Validation of ovarian cancer organoids against ovarian tumors in 3D and identifying cellular heterogeneity in intact tumors at single cell resolution. 3D evaluation of organoids can be advantageous for more accurate and rapid evaluation of drug response studies for personalized treatments in the future.
Cryopreservation of human ovarian tissue pieces aims to preserve fertility in patients.	What is the 3D composition of ovarian strips and what is the best cryopreservation protocol that maintains the tissue structure?	Mapping complex ovarian structures to advance our understanding of the human ovary and providing a valuable tool to compare the effects of different cryopreservation protocols on the 3D structural integrity of the ovarian follicles, vasculature, and stroma.
Follicle numbers and ovarian microenvironment (increased fibrosis, stiffness, and inflammation) change with aging.	How do the hallmarks of ovarian aging affect tissue structure?	Newly developed probes can provide better labeling for aged ovaries. 3D visualization can reveal complex vascular and neuronal network, and fine details of ovarian aging that potentially affects the function of the ovary.

*mESC, mouse embryonic stem cells; PGCLCs, primordial germ cell-like cells; rOvaries, reconstituted ovaries; 3D, three-dimensional.*

Insights gained from structural and functional 3D mapping of the ovary will aid in studying the progression and treatment of ovarian disease through novel organoid technologies. As *in vitro*-produced model organ structures containing several cell types of the target organ, organoids can be used to recapitulate tissue organization and structure, and to study cellular and tissue function in the context of diseases *in vitro* (Clevers, 2016). Although traditional 2D cell culture is the backbone of *in vitro* studies, development of 3D organoids more closely mimics the physiological composition of tissues and is gaining traction for understanding diseases and developing drugs as well as cell therapies. 3D ovarian cancer organoids were generated using patient-derived tumor cells and used to establish experimental models, perform drug response assays, and subsequently to develop personalized treatment strategies for ovarian cancers (Kopper et al., 2019; Maru et al., 2019; Nanki et al., 2020). Despite the efforts spent for their generation, these 3D ovary organoid structures were analyzed in 2D using conventional histological preparations. A direct comparison of the 3D structure of ovarian cancer organoids to tumors as well as ovary tissue would validate this model and potentially provide useful insights. More widespread adoption of techniques for clearing and 3D analysis will be important for improving ovarian cancer organoids, precisely mapping cell-cell interactions, and identifying cellular heterogeneity within tumors, particularly during drug responses (Table 2).

The application of 3D technologies to human ovaries will also augment current knowledge and practices of fertility preservation treatments. Cryopreservation of human ovarian cortical strips is becoming increasingly common as a means of preserving fertility. This procedure aims to protect fertility in cancer patients, particularly in pre-pubertal girls who lose their ovarian function as a result of chemotherapy or radiation (Wallace et al., 2014; Rivas Leonel et al., 2019). The success of fertility preservation treatment depends on the number of follicles maintained in cryopreserved tissue pieces, which is a function of the cryopreservation technique. The efficiency of cryopreservation has been compared by assessing follicle numbers and structure before and after using 2D conventional histology (Nisolle et al., 2000; Kagawa et al., 2009; Silber et al., 2010). With a different approach, Soleimani et al. (2006) took visualized human ovarian tissue strips in 3D after staining with a vital dye, Rhodamine 123 (R123) that would enable analysis without compromising transplantability. However, the absence of a clearing step in live tissue limits analysis to samples that are thinner than 0.5 mm. New developments in clearing techniques and 3D analysis of human ovary pieces will enable the mapping of complex ovarian structures such as follicles, vasculature and nerves at the single cell level, providing fundamentally new basic information about human ovarian follicle development. Careful examination of human cortical ovarian strips will also provide a valuable tool to compare the impacts of different cryopreservation protocols on the 3D structural integrity of the ovarian follicles, vasculature, and stroma in order to establish the least harmful protocol for clinical specimens (Table 2).

Although different clearing approaches greatly extend the limits of high-resolution analysis of intact organs (and even entire intact organisms), the field of 3D ovary modeling has adopted a limited number of these techniques. Despite the relative ease of clearing the fetal and adult ovaries, increased fibrosis and stiffness of the ovary with aging (Briley et al., 2016; Amargant et al., 2020) make the visualization and downstream analysis more challenging. To increase labeling efficiency and enhance subcellular protein identification in aged ovaries, recently developed probes and fluorophores provide promising alternatives to conventional immunoglobulin G (IgG) antibodies. Nanobodies are monomeric (heavy chain only) antibody fragments with the advantages of small size (15 kDa compared to 150 kDa for IgGs) allowing deeper and more effective penetration into tissue, higher binding affinity for their targets, and increased fluorescence intensity, thus providing better visualization of samples (de Beer and Giepmans, 2020). A nanobody based approach, along with an improved clearing protocol, was implemented in whole-body immunolabeling and visualization of mice, enabling Cai et al. (2019) to map neuronal projections in adult mice, and simultaneously to uncover short vascular connections in the brain which had previously not been visualized. Accordingly, improving the clearing and labeling efficiency of the aged ovary by applying these recent developments in the 3D modeling era will elucidate potential mechanisms contributing to the age-related decline in fertility as well as other pathologies (Table 2).

## Conclusion

The pioneering studies of the last 15 years paved the way for deciphering the 3D structure and function of the mammalian ovary. In the longer term, the development of non-toxic clearing reagents would enable 3D visualization of specimens for downstream analysis and manipulation in the lab or treatments in the clinic. With the evolution and widespread adoption and combination of tissue clearing technologies, imaging and analysis tools with other cutting edge genomic and machine learning technologies, the field will be poised to unravel the complexity of the mammalian ovary, and ultimately to advance female reproductive health and aging.

## Author Contributions

BS and DJL conceived and wrote the review. Both authors contributed to the article and approved the submitted version.

## Conflict of Interest

The authors declare that the research was conducted in the absence of any commercial or financial relationships that could be construed as a potential conflict of interest.

## Publisher’s Note

All claims expressed in this article are solely those of the authors and do not necessarily represent those of their affiliated organizations, or those of the publisher, the editors and the reviewers. Any product that may be evaluated in this article, or claim that may be made by its manufacturer, is not guaranteed or endorsed by the publisher.
